# Antimicrobial Activity of Blow Spun PLA/Gelatin Nanofibers Containing Green Synthesized Silver Nanoparticles against Wound Infection-Causing Bacteria

**DOI:** 10.3390/bioengineering9100518

**Published:** 2022-10-01

**Authors:** Elham Alinezhad Sardareh, Moloud Shahzeidi, Mohammad Taha Salmanifard Ardestani, Mohammad Mousavi-Khattat, Atefeh Zarepour, Ali Zarrabi

**Affiliations:** 1 Department of Biology, Nourdanesh Institute of Higher Education, Meymeh, Isfahan 83517-65851, Iran; 2 Department of Animal Biotechnology, Cell Science Research Center, Royan Institute for Biotechnology, ACECR, Isfahan 81593-58686, Iran; 3 Department of Biology, Faculty of Science, Yazd University, Yazd 89158-18411, Iran; 4 Department of Biotechnology, Faculty of Biological Science and Technology, University of Isfahan, Isfahan 81746-73441, Iran; 5 Department of Biomedical Engineering, Faculty of Engineering and Natural Sciences, Istinye University, Istanbul 34396, Turkey

**Keywords:** nanofibers, solution blow spinning, hemolysis, PLA, gelatin, antibacterial, cytotoxicity green Ag nanoparticles, cell viability

## Abstract

One of the main challenges in wound healing is the wound infection due to various causes, of which moisture is the most important reason. Owing to this fact, wound dressings that can collect wound moisture in addition to showing antibacterial properties have provided an important basis for wound healing research. In this study, gelatin and poly lactic acid (PLA) polymers were used in a wound dressing textile to provide gelation and structure strength properties, respectively. Meanwhile, silver nanoparticles (SNPs) synthesized through the green method were integrated into these fibers to provide the formed textile with antibacterial properties. Nanoparticles were made using donkey dung extract, and nanofibers were produced by the solution blow spinning method which has high production efficiency and low energy consumption among spinning methods. The produced nanoparticles were characterized and evaluated by UV-Vis, DLS, XRD, and FTIR methods, and the production of silver nanoparticles that were coated with metabolites in the extract was proven. In addition, the morphology and diameter of the resulted fibers and presence of nanoparticles were confirmed by the SEM method. The size and size distribution of the synthesized fibers were determined through analyzing SEM results. Gelatin nanofibers demonstrated a mean size of 743 nm before and 773 nm after nanoparticle coating. PLA nanofibers demonstrated a mean size of 57 nm before and 182 nm after nanoparticle coating. Finally, 335 nm was the mean diameter size of gelatin/PLA/SNPs nanofibers. Also, the textiles synthesized by PLA and gelatin which contained silver nanoparticles showed higher antibacterial activity against both gram-positive and gram-negative species compared to PLA and gelatin tissues without nanoparticles. Cytotoxicity test on L929 cells showed that silver nanoparticles incorporated textiles of PLA and gelatin show a very low level and non-significant toxicity compared to the free particles.

## 1. Introduction

Nanotechnology is the manipulation of nanoscale materials in various fields such as engineering, materials science, chemistry, and biology [1,2,3,4]. This area of research has been compared to the industrial revolution as one of the most essential aspects of science and technology in the twenty-first century [5,6]. Nanomaterials are, in principle, materials in which a single unit small-sized (in at least one dimension) between 1 and 100 nm has unique properties such as small size and surface-to-volume ratio in comparison with their bulk components, which provide specific light or have magnetic, surface, and electrical or chemical properties. Potential applications of nanomaterials in many industrial sectors including biomedicine, defense, energy and storage, pharmacy, food, agricultural reform, and the environment have been confirmed [7].

Nanofibers are an interesting group of nanomaterials with two similar external dimensions at the nanoscale, and the third dimension is considerably larger [8]. These nanostructures are good candidates for a variety of biomedical applications, including tissue-engineered scaffolds (e.g., skin, cartilage, bone, and blood vessel), biomedical devices, biosensors, drug delivery systems, and wound dressings [9,10,11,12,13]. These nanostructures have high interconnected porosity (60–90%), high absorbance, balanced moisture, and gas permeability, all of which offer a suitable environment for wound protection against external infection [14].

Wound healing has been a major health problem and a global medical concern. The process involves several stages including homeostasis, inflammation, proliferation, cell migration, and finally tissue regeneration. Advanced wound dressing plays an important role in surface protection, control of bacteriostatic activity, moisture retention, and shortening of tissue recovery period due to its excellent wound dressing properties [15,16]. The main role of wound dressing includes maintaining wound hydration, absorbing excess wound secretions, minimizing the impact on the wound site, and as a barrier to the entry of foreign microorganisms into the wound site [17,18,19]. The nanofiber dressings as a product of nanotechnology were designed to decrease infection, irritation and improve wound healing by providing a positive environment in addition to their usual duties [20,21]. In comparison with macro-scale dressings, nanofibers contain more moisture in their structure and keep the wound area moist during the healing process. This inhibits the nanofibers from adhering to the wound surface. Furthermore, the porous nanofiber network facilitates oxygen diffusion into the wound surface [22].

There are different methods for nanofiber design and fabrication such as force spinning, charge injection spinning, gas-assisted spinning, bubbfil spinning, and electrospinning [23]. Electrospinning as one of the most common techniques in wound dressing could produce biological nanofibers from a wide range of natural and synthetic polymers with biologically related properties. However, the electrospinning method has disadvantages such as low performance, high operating voltage, and difficulty in depositing nanofibers on different substrates [24]. Solution blow spinning (SBS) as an alternative is a new technique for producing micro- or nano-scale fibers. SBS was first reported by Medeiros et al. in 2009 [25]. This technology produces nanofibers from a polymer solution through the use of pressurized gas. Nanofibers are formed from parallel streams of the polymer solution and pressurized gas (like air or nitrogen) that are blown through concentric chambers of a nozzle. The polymer solution is in the inner chamber while the pressurized gas is in the outer chamber. The pressurized high-velocity gas causes a pressure drop and shearing at the gas/solution interface resulting in the stretching of the polymer solution towards a fixed collector. The streams of stretched polymers then rapidly form into fibers as the solvent evaporates. The resulting nanofibers show attractive properties such as high specific surface area, high porosity, and flexibility in surface operations and due to these unique features, it is considered a desirable option for wound dressing purposes. Also, a wide range of polymers can be used in the fabrication of nanofibers for various applications such as tissue engineering, drug delivery, filtration, and so on [26]. Another important feature of the ideal wound dressing is Its antimicrobial property. The infection in the wound can slow down the wound healing and cause the infection to spread to the rest of the body [27]. The antibacterial property of wound dressings can be achieved by using polymers with antimicrobial properties or by adding drugs and materials with antibacterial properties to the wound dressing [28]. In the meantime, silver nanoparticles have received much attention because they do not create bacterial resistance and are used in some wound dressings [29]. On the other hand, one of the most important issues regarding wound dressings is their biocompatibility in dealing with blood cells. Because wound dressings are generally in direct contact with blood flow and can challenge blood cells. The toxic effect of these wound dressings on the normal cells of the body is also important, so that it does not cause problems in wound healing and does not prevent the growth of skin cells [30,31].

The main goal of this study is to produce nanofibers with the SBS technique containing antimicrobial nanoparticles for wound healing purposes. Silver nanoparticles (AgNPs) have been used as an antimicrobial agent against a wide range of pathogenic microorganisms and have also been combined with degradable polymers to maintain the stability and antibacterial activity that is most needed [32]. The silver nanoparticles synthesized by the green method were loaded on gelatin and PLA nanofibers fabricated through the SBS technique. The use of biodegradable polymers with a volatile solvent makes the structure non-toxic and solvent free for medical uses. Gelatin improves the capacity of wound dressings to absorb water and keep the surroundings wet.

## 2. Materials and Methods

Poly (lactic acid) (PLA, Mw = 75,000 g/mol) pellets, ethylenediaminetetraacetic acid (EDTA), dimethylsulfoxide (DMSO), dichloromethane (CH_2_Cl_2_), acetic acid aqueous solution (CH_3_COOH, wt = 10%) and silver nitrate (AgNO_3_) were purchased from Merck Co. (Frankfurter, Germany). Gelatin prepared from Helal sahand (Tabriz, Iran). 3-(4,5-Dimethylthiazol-2-yl) (MTT), Dulbecco’s Modified Eagle Medium (DMEM) cell culture medium, penicillin-streptomycin (Pen-Strep), phosphate-buffered saline (PBS) Trypsin enzyme and fetal bovine serum (FBS) were all bought from Gibco Co. (New York, NY, USA). Nutrient agar was bought from Quelab Co. (Montréal, QC, Canada). *Escherichia coli* and *Staphylococcus aureus* strains were obtained from the Iranian Biological Resource Center (Tehran, Iran). The L929 cell line was purchased from Royan Institute for Biotechnology (Isfahan, Iran).

### 2.1. Green Synthesis of Silver Nanoparticles

Synthesis of silver nanoparticles was performed using medicinal donkey dung (Anbarnesa) extract as a green reducing/stabilizing agent according to our previous study [33]. As an extraction method, 50 mL of distilled water was added to the 5 g of donkey dung dried powder and the mixture was stirred at 70 °C for 15 min. Then the solution was cooled at room temperature and filtered. The brownish extract of animal waste was freshly used to synthesize the nanoparticles. Then, the pH of the solution was set, and 2 mL of animal waste extract was added to 100 mL of AgNO_3_ (1 mM). The mixture was stirred for 2 h at 70 °C and then freeze-dried for further analysis.

### 2.2. Nanoparticle Characterization

From the start of the reaction over 60 min with a time interval of 10 min between 300 and 600 nm, UV–Vis spectral analysis was performed using the UV–Vis model Biowave II, UK for green synthesized silver nanoparticles (GS-SNPs). At room temperature, two milliliters of each sample were pipetted into a cuvette and examined. The average size of produced silver nanoparticles was determined using dynamic light scattering (VASCO™, Cordouan-tech, Pessac, France). The surface charges and stability of the nanoparticles in suspension of resulted particle was determined through a Zeta-sizer Instrument (SZ-100, Horiba, Kyoto, Japan). On the FT-IR (Fourier-transform infrared spectroscopy) Spectrometer 6300, Jasco, Tokyo, Japan, FT–IR spectra were obtained. The dried and ground AgNPs sample were analyzed for several modes of vibration to validate the presence of functional groups in AgNPs. To check the silver content of produced nanoparticles, the powdered nanoparticles were placed in an X-ray diffractometer (D8 Advanced X-ray diffractometer, Bruker, Billerica, MA, USA) with a wavelength of 1.540598 °A (Cu K). Non-diluted AgNP samples were placed on a grid for transmission electron microscopy (TEM) to check the shape and diameter of the nanoparticles (Zeiss-EM10C-100 KV, Oberkochen, Germany).

### 2.3. Nanofiber Fabrication

In this process, 0.4 g of poly (lactic acid) was dissolved in dichloromethane in a total volume of 10 mL to make a 4% (*w*/*v*) solution. A 24% (*w*/*v*) concentration of gelatin was obtained by dissolving 2.4 g of it in 10 mL of acetic acid. Due to the biphasic nature of the solutions when they mix together, each one was mixed and spun separately. The PLA solution was spun through SBS method with an air pressure of 0.5 bar and a flow rate of 100 µL/min for 10 min. The solution was pushed out through the hole of the needle using air pressure so that after the evaporation of the solvent, it is placed on the rotating collector in the form of fiber. The obtained textile was given time to completely evaporate the solvent to prepare a suitable substrate for spinning the second polymer. On the other hand, GS-SNPs were also added to the solution containing gelatin (second polymer) to improve their release properties due to the gelling properties of gelatin. Thus, 0.2 g of GS-SNPs was mixed gently with 10 mL of gelatin/acetic acid polymer solution to achieve 2% *w*/*v* of GS-SNPs/gelatin solution. All of these saluting and mixing processes were performed for 24 h under magnetic stirring at room temperature. Finally, GS-SNPs/gelatin solution was spun upon PLA textile through SBS method with an air pressure of 0.5 bar and a flow rate of 100 µL/min for 10 min.

### 2.4. SEM Analysis

Morphological analysis of GS-SNPs and nanofibers presented through SEM (Philips XI30 Electron Microscopy, Eindhoven, The Netherlands). Before the observation, the mats were coated with gold using a sputter coater (Technics, Hummer II, Haarlem, The Netherlands) and imaged at 15 kV. The diameter distribution of the fibers for each sample was measured using ImageJ software.

### 2.5. Nanoparticle Release Measurement

The release profile of SNPs from PLA/SNPs, Gel/SNPs and PLA/Gel/SNPs textiles were analyzed by dispersing the textiles (50 mg) in a PBS buffer. The suspensions were stirred at a predetermined time (0.5–24 h) at 37 °C, followed by centrifuging and measuring the SNPs content by a microplate reader at 450 nm (800TS, Biotek, Santa Clara, CA, USA). Also as reference Ag+ samples for a calibration curve, silver nitrate solutions in a range of concentrations (0.0, 5.0, 7.5, 10, 20, and 30 × 10^−4^ mM) were generated [34]. Drug release data was evaluated using different kinetic models, such as Korsmeyer Peppas, zero order, first order, Higuchi, and Hixon-Crowell to confirm the mechanism and kinetics of drug release. The value of R^2^ from the linear regression equation generated from each calculation was used to determine the drug’s release kinetics. One can assume that the kinetic followed the release equation from the relevant kinetics model if R^2^ was close to one [35].

### 2.6. Hemolysis Test

Blood was obtained from a healthy human volunteer and was anticoagulated with EDTA then centrifuge for 5 min with 3900 rpm. RBCs were washed by centrifugation and decanting five times with PBS (Cellular condensate was diluted by PBS to prepare cellular suspension with 100 million cells per mL. 400 µL of current suspension was added to each pit in 48 well-plates. Final textures in different concentrations were added for each well. One well was left blank with no cellular suspension and another one was filled by cell suspension with no treatment as standard. Triton X-100 was used as positive control. Finally, the plate was incubated for 4 h at 37 °C within CO_2_ atmosphere.

### 2.7. Cytotoxicity 

Cell culture: L929 fibroblast cells from Isfahan University of Medical Sciences, Isfahan, Iran, were cultured in 96-well plates with 1 × 10^5^ cells per well DMEM culture media containing 10% FBS and 1% Pen-Strep (100 U/mL Penicillin and 100 g/mL Streptomycin) (5 % CO_2_ at 37 °C).

The MTT assay was used to assess the nanofibers potential cytotoxicity. Due to the high hydrophilicity of the gelatin, UV light was used to sterilize the tissues so that each side of the tissue was exposed to UV light for 45 min [36]. The sterile nanofibers were separately placed on a plate with DMEM medium and incubated for 24 h (at 37 °C and 5% CO_2_). Then, media extraction samples were taken after 24 h and the cell culture medium was removed, the extracts were replaced, and 10% fetal bovine serum was added to the extraction medium. The cells underwent a second 24-h incubation. Finally, the cells were cultured for 4 h in 100 µL of media containing 10% MTT solution (5 mg/mL). The formazan crystals created in the living cells were dissolved in 100 µL of DMSO after the media was withdrawn. Using a microplate reader, the absorbance at 575 nm was determined to calculate the percentage of cell viability. Cells exposed to fresh media devoid of nanofiber was used as negative control [36].

### 2.8. Antibacterial Test

The standard strains of *Escherichia coli (E. coli)* and *Staphylococcus aureus (S. aureus)* were used in this work. The bacteria were suspended in PBS, and the concentration of bacterial suspension was adjusted by a spectrophotometer at 600 nm. The final concentration of prepared *E. coli* and *S. aureus* medium was about 1.5 × 10^8^ per ml. The antimicrobial properties of each textile was tested on nutrient agar plates inoculated with *E. coli* and *S. aureus* strains through disk diffusion method [37]. After 24 h of incubation, inhibition zones around PLA/gelatin textiles without and with SNPs were evaluated. In order to perform bacterial quantification test, samples were examined in different groups. For this purpose, the samples were placed in a 12-well plate and 400 µL of *S. aureus* and *E. coli* bacteria were diluted with PBS buffer to achieve a concentration of 1.5 × 10^8^ bacteria ml-1 and the plate was incubated at 37 °C for 12 h. Then, 100 µL of the surface solution was transferred into a 96-well plate to read their absorbance at 600 nm. Also, 20 µL of the surface solution of each well was spread on a nutrient agar plate and placed in a 37 °C incubator for 24 h. After this time, the colonies were counted on each of the plates by ImageJ software and their number was compared [38].

## 3. Results

### 3.1. Synthesis and Characterization of Silver Nanoparticles

**Figure 1:**
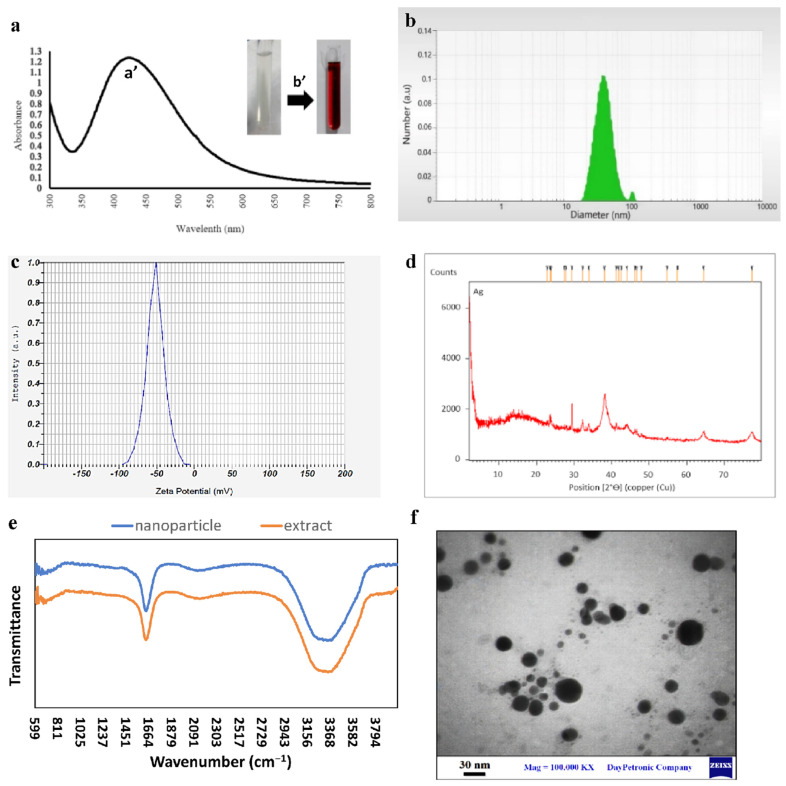
(**a**): UV-Vis spectroscopy of green synthesized SNPs (**a’**). color change after the reaction was completed (**b’**). (**b**): DLS results of green synthesized SNPs. (**c**): Zeta potential of green synthesized SNPs. (**d**): XRD analysis of green synthesized SNPs. (**e**): FTIR analysis of the extract and the SNPs with which synthesized. (**f**): Microscopic study of green synthesized SNPs by TEM.

In this study, after adding Anbarnesa extract to silver nitrate solution under defined conditions, the color changes from clear to dark brown as the first sign of the formation of the nanoparticles. Also, nanoparticles after examination by spectrophotometer showed a narrow peak in the wavelength range of 425 nm, which is typical for the maximum UV absorption of silver nanoparticles (Figure 1a). The results of DLS analysis also showed that the nanoparticles were at the nanometer scale and their diameter is generally less than 100 nm (Figure 1b). Their average diameter was estimated at 28.17 nm. As an index of stability, Zeta potential of the synthesized SNPs was measured and a negative value of −51.7 mV demonstrated high stability of the SNPs (Figure 1c) [39]. The XRD spectrum pattern of silver nanoparticles (Figure 1d) shows that there is silver metal in our solution because the indicator peaks (38.08, 46,69, 64.52, 77.34) at 2θ° are special silver peaks. The FTIR spectrum of donkey dung extract and green synthesized AgNPs using donkey dung were analyzed to confirm the phytochemical coating of the nanoparticles during the synthesis process indicated in Figure 1e. The results showed that the presence of absorption peaks at 3339 cm^−1^ and 2130 cm^−1^ and 1637 cm^−1^ are related to phenolic compounds, C–H stretching bonds, and carbonyl groups respectively. Images obtained by microscopic analysis by TEM showed that the nanoparticles were spherical in shape and nanoscale in size. Therefore, the DLS results were confirmed (Figure 1f).

### 3.2. Characterization of Nanofiber Mats

**Figure 2:**
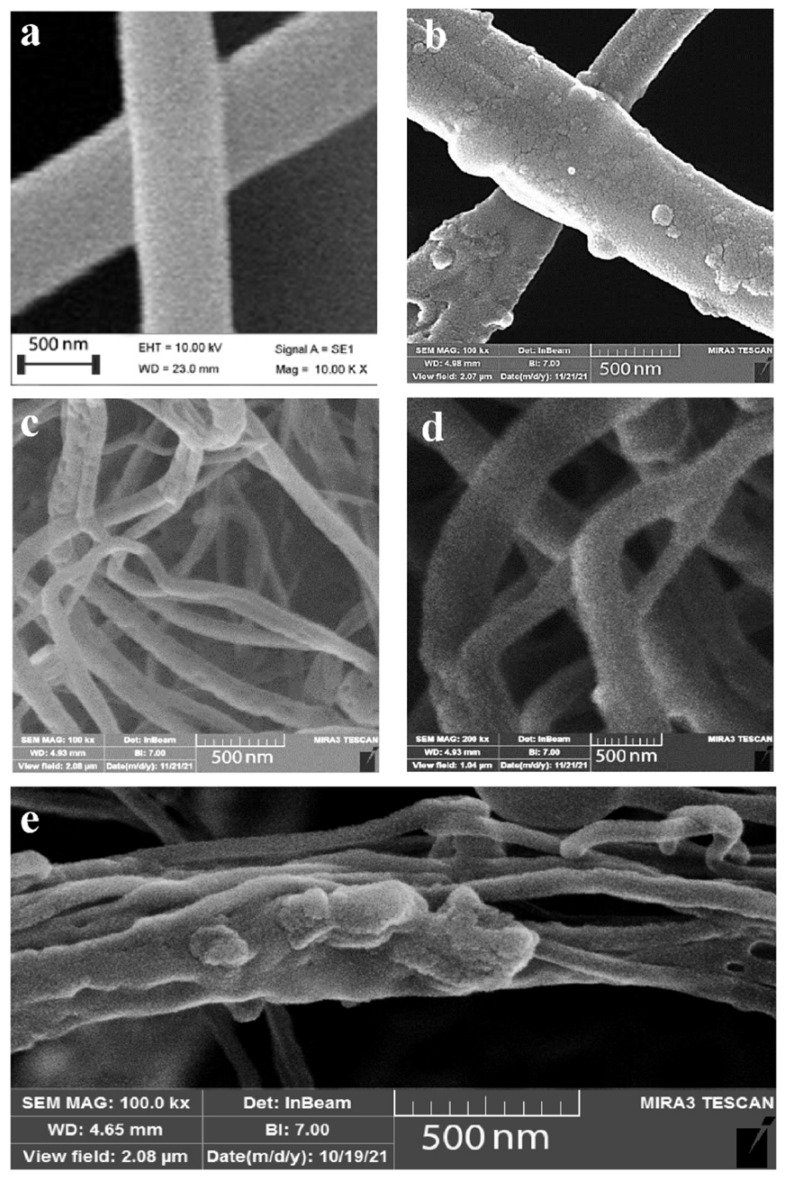
Blow spun nanofibers by gelatin (**a**), Gel/SNPs (**b**), PLA (**c**), PLA/SNPs (**d**) and PLA/Gel /NPs (**e**) solutions.

**Figure 3:**
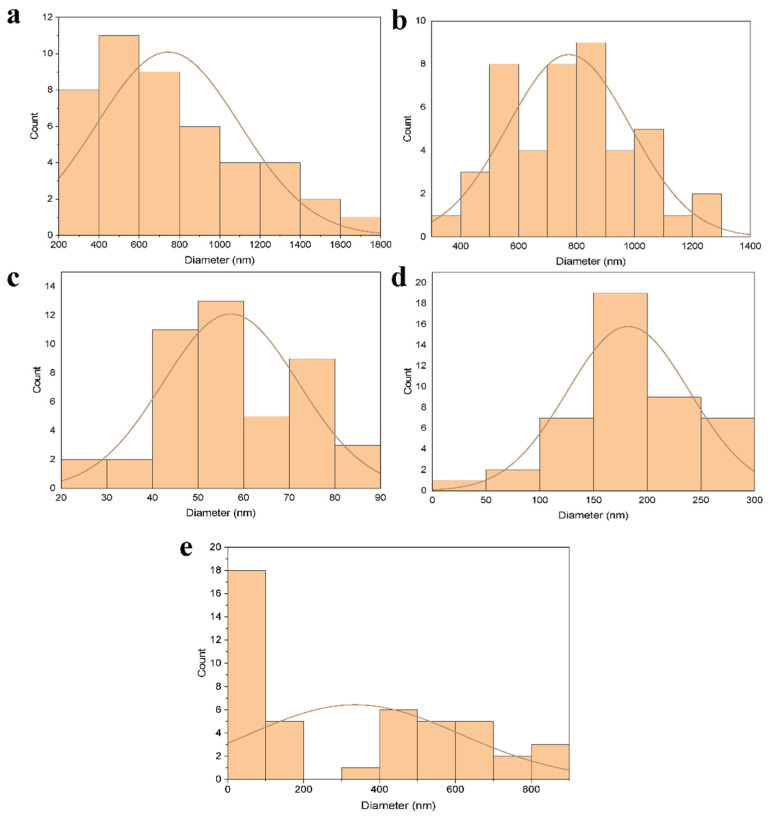
(**a**): Size distribution of gelatin nanofibers before coating with nanoparticles. (**b**): Size distribution of gelatin nanofibers after coating with nanoparticles. (**c**): Size distribution of PLA nanofibers before coating with nanoparticles. (**d**): Size distribution of PLA nanofibers after coating with nanoparticles. (**e**): Size distribution of gelatin/PLA nanofibers blended with nanoparticles.

**Figure 4:**
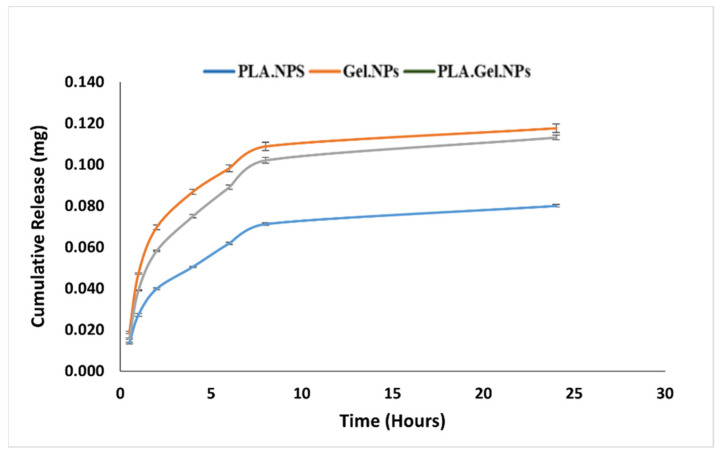
SNPs release profiles from different textiles.

After optimizing the concentration of polymer and silver nanoparticles as well as air pressure, the obtained films that were stacked in a thin layer were studied by SEM to determine the morphology of the produced films and their diameter size. The SEM results show that nanofibers spun with gelatin alone (Figure 2a) have a higher smoothness than nanofibers made with a mixture of gelatin and nanoparticles (Figure 2b) due to the presence of nanoparticles on the surface of the fibers. In both cases, the fibers are stacked in random directions and their size is in the range of 200 to 700 nm. Similar to gelatin/SNPs nanofibers for the fabrication of fibers by PLA alone and with the presence of nanoparticles (Figure 2c,d respectively), the surfaces of the nanofibers formed by the second solution were rough and it seems that nanofibers are closer than gelatin fibers with a partially random direction. Figure 2e. shows the SEM results for spinning polymers gelatin and PLA with silver nanoparticles. The rough surface of the nanofibers indicates the presence of silver nanoparticles on the surface. Figure 3 shows the size distribution of gelatin and PLA nanofibers without (Figure 3a,c) and with (Figure 3b,d) silver nanoparticles obtained through ImageJ analysis. Also, a size distribution of gelatin/PLA/SNPs is demonstrated in Figure 3e. The mean size of gelatin nanofibers before and after nanoparticle coating was 743 nm and 773 nm, respectively. Using the same methodology, PLA nanofibers showed a mean size of 57 nm prior to nanoparticle coating and 182 nm afterwards. Also, the mean diameter size of the gelatin/PLA/SNPs nanofibers was 335 nm. Based on results provided from SNPs release profile embedded in textiles it was shown that Gel/SNPs and PLA/Gel/SNPs nanofibers have higher release rates in contrast with PLA/SNPs having low rate of SNPs release (Figure 4). Mathematical model results indicate that the formulation appears to follow zero-order models because it has the highest R^2^ value (Table 1).

### 3.3. Blood Compatibility Test

**Figure 5:**
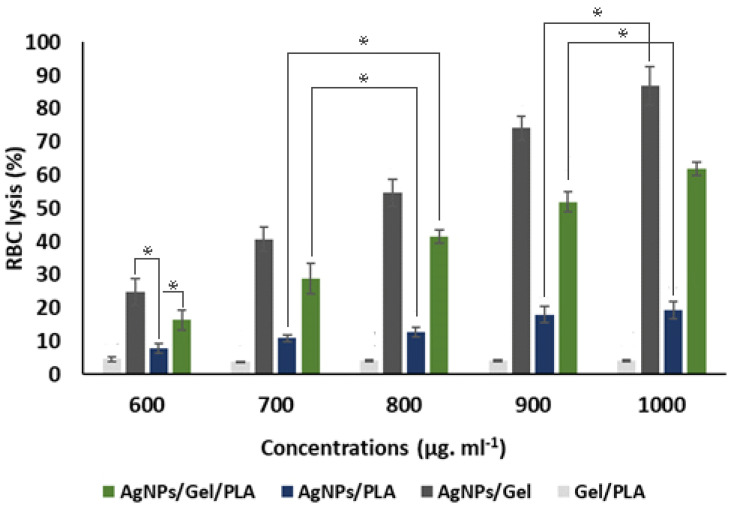
RBC lysis activity of Gel/PLA, AgNPs/Gel, AgNPs/PLA and AgNPs/Gel/PLA mats for different textiles concentrations (600 μg.mL^−1^–1000 μg.mL^−1^) using PBMC cells. The experiments were repeated in triplicate and the control sample was free PBS, which showed zero% RBC lysis. Asterisks indicate statistically significant differences using Duncan’s test (* *p* < 0.05).

Blood compatibility is a feature that all biomaterials that come into touch with blood elements should have. The potential for hemolysis caused by the manufactured nanofibrous wound dressing materials was assessed in this study as a sign of blood compatibility. The hemolysis activity decreased in the following order respectively; Gel/SNPs, PLA/Gel/SNPs, PLA/SNPs, and PLA/Gel synthesized nanofibers (Figure 5)

### 3.4. Cytotoxicity Test

**Figure 6:**
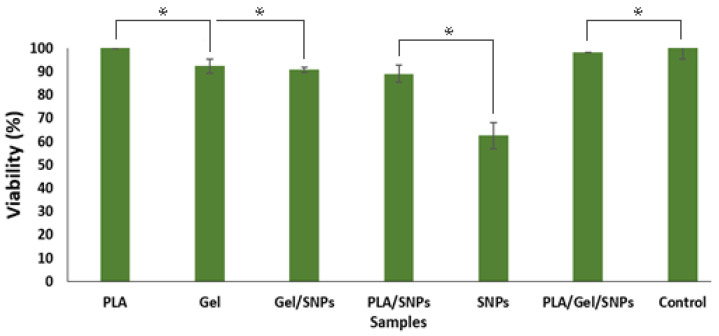
Viability (%) of L929 mouse fibroblast cells by Indirect MTT assay of different mats over 24 h of culture. Control: Cells and culture media without any textiles. The experiments were repeated in triplicate. Asterisks indicate statistically significant differences using Duncan’s test (* *p* < 0.05).

**Figure 7:**
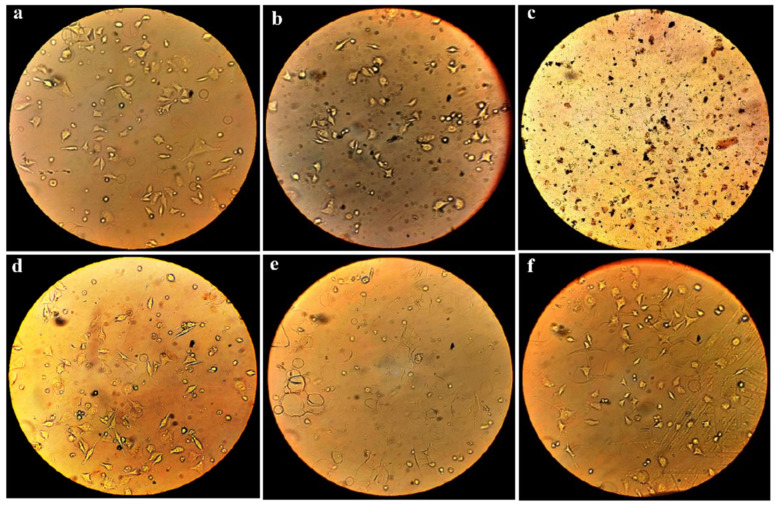
Microscopic images of cultured L929 cells on different media containing PBS as control (**a**) PLA/Gel (**b**), SNPs (**c**), PLA/SNPs (**d**), Gel/SNPs (**e**), and PLA/Gel/SNPs after 24 h (**f**). Transparent multi-branched objects are alive and healthy cells, spherical ones are low proliferated cells and dark spots are dead cells.

Cell viability and proliferation were assessed using the MTT assay to compare the number of cells in the free SNPs and different synthesized nanofiber mats. Cells and culture media without any textiles were used as a control. The results showed that silver nanoparticles in their free state show the most toxicity against normal cells, but if these nanoparticles are trapped in the nanofibrous networks, they induce a lower level of toxicity (Figure 6). The MTT assay relies on the dehydrogenase enzymes produced from the mitochondria of metabolically active cells to convert the yellow tetrazolium salt into purple formazan crystals. The quantity of live cells is inversely correlated with the amount of purple formazan crystals produced. Microscopic images of each sample are represented in Figure 7. Cells that are alive and healthy are spindle-shaped or multi-branched in microscopic images (Figure 7a,b,d,f), but cells that have not multiplied well are spherical (Figure 7e), and dead cells are seen as dark spots (Figure 7c).

### 3.5. Antibacterial Activity

**Figure 8:**
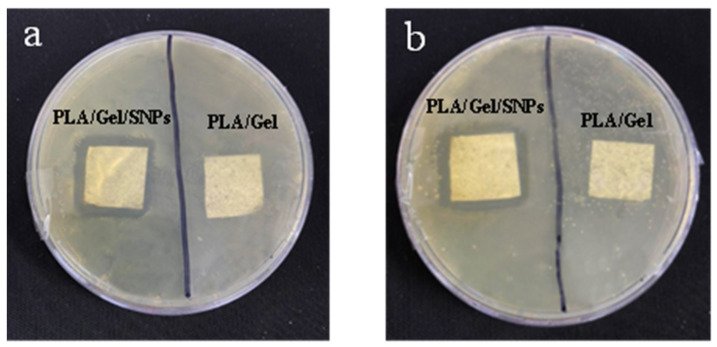
(**a**): Diffusion test for antibacterial activity of PLA/Gel/SNPs and PLA/Gel on *S. aureus*. (**b**): Diffusion test for antibacterial activity of PLA/Gel/SNPs and PLA/Gel on *E. coli*.

**Figure 9:**
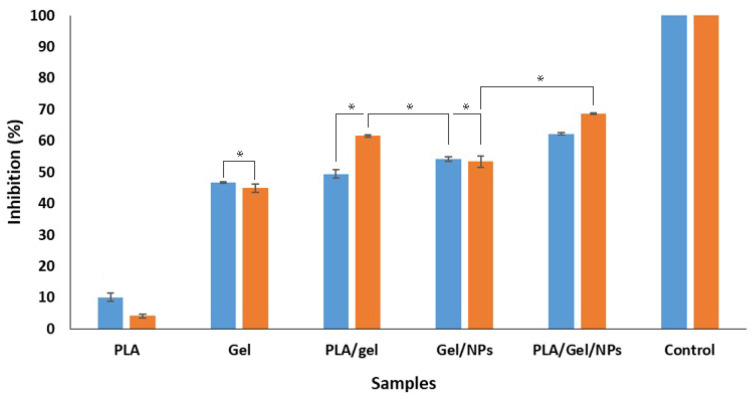
Quantitative antibacterial activity of different synthesized mats on *E. coli* (blue) and *S. aureus* (orange). Control: Bacteria and culture media without any textiles. The experiments were repeated in triplicate. Asterisks indicate statistically significant differences using Duncan’s test (* *p* < 0.05).

**Figure 10:**
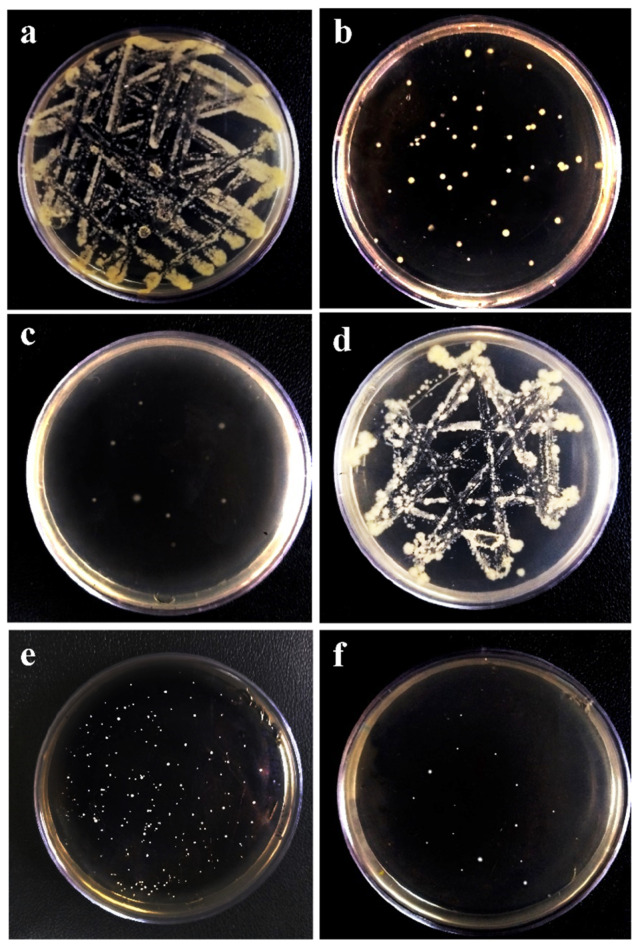
The colony culture results of different samples: (**a**): Negative control for *E. coli* (Bacteria and culture media without any textiles), (**b**): PLA/Gel on *E. coli*, (**c**): PLA/Gel/SNPs on *E. coli*, (**d**): Negative control for *S. aureus* (Bacteria and culture media without any textiles), (**e**): PLA/Gel on *S. aureus*, (**f**): PLA/Gel/SNPs on *S. aureus*.

The disk diffusion method was used to investigate the antibacterial activity of PLA/Gel/SNPs mats against *S. aureus* and *E. coli*, as the typical species of gram-positive and gram-negative bacteria respectively. For PLA/Gel/SNPs spun nanofibers the inhibition zone diameters for both bacteria were similar (about 5 mm from the edge of each side) and No zone was seen for the nanofibers that lacked nanoparticles (Figure 8). Quantitative analysis of nanofibers antibacterial activities demonstrated in Figure 9. The results show almost nothing antibacterial activity for PLA nanofibers when used alone in contrast with gelatin which has a little antibacterial effect as demonstrated in previous studies [40]. After PLA is combined with Gel, their antibacterial effect is gathered together and they show more antibacterial effect, but when silver nanoparticles are added to Gel, we see a further increase in the antimicrobial properties of the tissue. Finally, the combination of PLA/Gel/SNPs has the most antibacterial properties during overnight. Bacteria and culture media without any textiles were used as a control. The number of colonies counted after sampling the bacteria found in different samples and spreading it on agar plates, also shows that after adding silver nanoparticles to nanofibers, their antibacterial properties have increased significantly (Figure 10). *E. coli* cultures in the presence of PLA/Gel and PLA/Gel/SNPs showed 46 and 10 colonies on the plates and for *S. aureus* the amounts of 101 and 17 were demonstrated respectively. Bacteria and culture media without any textiles were used as the control.

## 4. Discussions

Several studies have been published on the fabrication of nanofibers and their use in wound dressings. However, the use of nanoparticles with antibacterial properties in nanofiber wound dressings is a new field in the production of antibacterial nanofibers that has been less studied [41,42]. In this study, in addition to using two types of polymers with different properties, green synthesized silver nanoparticles with antimicrobial properties synthesized by donkey dung extract were used to increase antimicrobial properties with the lowest toxicity. The antibacterial effect of silver nanoparticles made from biological sources has already been investigated and has shown good efficacy against gram-positive and gram-negative infections [33,43]. Due to the presence of antibacterial substances in the extracts of donkey dung, in addition to their antibacterial properties, these nanoparticles also have the antibacterial properties of these metabolites and will increase their antibacterial properties. On the other hand, gelatin nanofibers have already been used in various wound dressings, the main reason being their gelatinizing properties. This property can cause the gradual release of the drug or active substance contained in them [44]. Moreover, gelatin nanofibers tend to mimic the structure and function of the extracellular matrix (ECM), having a protein fibrous network that gives structural support to surrounding cells [45]. Also, the reason for using PLA is to create sufficient strength along with gelatin due to the dissolution of gelatin in aqueous media. PLA can maintain the wound structure after absorbing secretions in the wound area. The release of silver nanoparticles by gelatin, which is carried out at a high rate compared to PLA, is the reason for using gelatin as a scaffold for silver nanoparticles. Also, the release kinetics in this study followed a zero order model based on the results. By releasing nanoparticles at a consistent pace and keeping concentrations within the therapeutic window for a longer period of time, zero-order drug delivery systems offer the potential to solve problems with immediate-release and first-order systems [46]. The biocompatibility of both polymers used in this structure and their non-toxicity had already been proven. The reduced toxicity of green nanoparticles produced by the green method has also been previously investigated [47,48]. However, in the present study, a cytotoxicity test by using different synthesized textiles was performed on L929 cells, and except for its high concentrations, no significant toxicity was observed at low and medium concentrations. The microscopic images obtained from the morphology of the cells also show the change of the spindle-shaped and transparent state that happens in the case of living and healthy cells to the spherical and dark state when the SNPs are alone or together with gelatin [49]. If there is PLA in the samples, it reduces the toxicity of SNPs, which can be due to maintaining the structure of the tissue and thus keeping it from dissolving and quickly releasing SNPs [50]. The time required for spinning polymers in previous studies was generally above one hour. This is despite the fact that gelatin and PLA polymers were spun in 10 min in the present study and the desired textiles were obtained [51,52]. The antibacterial test showed that silver nanoparticles, in addition to being able to exert their effect in the open environment, can also express their antibacterial properties among nanofibers and prevent the growth of bacteria. Overall, the combination of green nanoparticles made with green nanofibers, which are suitable both in terms of gelatinization and strength, can create promising horizons in the treatment of wound infections. Also, due to the low toxicity of this structure to normal cells, their use as a wound dressing can greatly reduce concerns about the toxicity of these structures.

## 5. Conclusions

Since one of the most important problems in the treatment of wounds is their infection and this infection is accompanied by secretion, the use of wound dressings that show antibacterial properties while absorbing moisture will be considered important. Since nanofibers alone do not have antibacterial properties, it is necessary to add substances that give them this property for disinfection purposes [53]. In this study, nanoparticles made by green method using plant metabolites with antibacterial properties as well as two nanofibers PLA and gelatin were used as wound dressing mats with antibacterial properties. Silver nanoparticles made by the green method, in addition to being able to bring the antibacterial properties of the extract with which they are made, have very low toxicity compared to the nanoparticles made by the chemical method and can be used as antibacterial substances in Wound. Also, nanofiber structure synthesized in the present study can absorb the secretions by using the gelatinizing property of gelatin and over time the gelatin changes from fiber to soluble, which will cause the gradual release of nanoparticles. These nanoparticles are then distributed in the wound environment and show their antibacterial properties to reduce wound infection and collect secretions. The zero-order release profile obtained in the present study can be used to limit adverse side effects, reduce dosing frequency, and potentially improve patient compliance. Given that both polymers PLA and gelatin were produced using the solution blow spinning method and each of them showed their suitable properties for use in wound dressing, we can expect the spraying method as an alternative to previous methods such as electrospinning. The electrospinning method consumes high cost, time, and energy to make nanofiber fabrics on a large scale, while the solution blow spinning method can be a suitable alternative for large-scale production by creating nanofibers with the same diameter with lower cost, time, and energy [48].

## Figures and Tables

**Table 1:** R^2^ values for drug release kinetic models.

Formulation/Model	Kors-Peppas	Zero Order	First Order	Higuchi	Hixson
PLA.NPs	R² = 0.8987	R² = 0.996	R² = 0.9913	R² = 0.9401	R² = 0.995
Gel.NPs	R² = 0.9165	R² = 0.9946	R² = 0.9637	R² = 0.9402	R² = 0.9856
PLA.Gel.NPs	R² = 0.9027	R² = 0.9965	R² = 0.9705	R² = 0.9307	R² = 0.9861

## Data Availability

The data presented in this study are available on request from the corresponding author.
